# Health-related quality of life among postpartum women with preeclampsia, southern Ethiopia: a prospective cohort study

**DOI:** 10.1186/s12955-022-02061-2

**Published:** 2022-10-29

**Authors:** Birhanu Jikamo, Mulat Adefris, Telake Azale, Kassahun Alemu

**Affiliations:** 1 Department of Epidemiology and Biostatistics, Institute of Public Health, College of Medicine and Health Sciences, University of Gondar, Gondar, Ethiopia; 2 Hawassa University College of Medicine and Health Sciences, Hawassa, Southern Nations Ethiopia; 3 Department of Gynecology and Obstetrics, School of Medicine and Health Sciences, University of Gondar, Gondar, Ethiopia; 4 Department of Health Education and Behavioral Sciences, Institute of Public Health, College of Medicine and Health Sciences, University of Gondar, Gondar, Ethiopia

**Keywords:** Pregnancy, Health-related quality of life, Preeclampsia, Normotensive, Ethiopia

## Abstract

**Background:**

Preeclampsia affects the health of the mother and the fetus during pregnancy and childbirth. To date, little is known about the impact of preeclampsia on postpartum health-related to quality of life (HRQoL) in the Sidama region of southern Ethiopia. This study aimed to measure the HRQoL and its contributing factors among postpartum women with preeclampsia in the Sidama region.

**Methods:**

A prospective cohort study was conducted by enrolling pregnant women at ≥20 weeks of gestation up until the 37th week of gestation. We then followed them until 12 weeks after delivery. A locally validated, World Health Organization Quality-of-Life-BREF (WHOQOL-BREF) tool was used to assess participants’ HRQoL at two time points; the 6th and 12th weeks postpartum. Assessment of HRQoL of participants was based on total scores on the WHOQoL-BREF. Higher scores on the WHOQoL-BREF reflected a higher HRQoL. Multiple linear regression analyses were performed to evaluate the contributing factors to HRQoL. The level of significance was determined at a *p-*value of < 0.05.

**Results:**

The HRQoL of postpartum women with preeclampsia significantly improved over time from 6 (151 ± 17) to 12 weeks (167 ± 18), *p <* 0.001). However, the overall HRQoL scores were lower (156 ± 16, *p <* 0.001) among women with preeclampsia compared to normotensive women (181 ± 21). An experience of early neonatal death was found to have a significant negative effect on the HRQoL of women with preeclampsia [β = − 2.1, 95% CI: − 3.43– − 0.85] compared to normotensive women who did not have early neonatal death. At 6 weeks of the postpartum period, the physical domain was found to have a significantly higher contribution to the lower HRQoL [β = 1.04, 95% CI: 0.88–1.12] compared to normotensive women, while other factors were constant.

**Conclusions:**

The HRQoL of women with preeclampsia improved over time from 6 to 12 weeks in the postpartum period. Lower HRQoL was observed among postpartum women with preeclampsia, especially among those who experienced preterm birth or early neonatal death. The effects of preeclampsia on the HRQoL of postpartum women should be considered in redesigning postnatal care intervention services.

**Supplementary Information:**

The online version contains supplementary material available at 10.1186/s12955-022-02061-2.

## Background

Preeclampsia is defined as the presence of proteinuria (≥1+ or 0.3 g/L) and hypertension (≥140/90 mmHg) on two occasions, at least 4 hours apart, detected after the 20th week of gestation in a previously normotensive woman [1]. What is more, preeclampsia is a placenta-related hypertensive pregnancy complication that affects 2–8% of pregnancies and is associated with both maternal and neonatal complications [1].

The postpartum period is a critical period characterized by physiological and psychosocial changes in postpartum women [2, 3]. Postpartum maternal morbidity after a preeclamptic pregnancy may be related to the multi-organ involvement of the disease and poor mental health that leads to a delay in early treatment [2, 3]. Women who have severe preeclampsia are often admitted to the intensive care unit, which may also influence psychological outcomes, especially when it develops early in pregnancy or when an adverse perinatal outcome occurs [2, 3]. Furthermore, women with severe preeclampsia reported more postpartum depressive symptoms at 6 and 12 weeks postpartum, compared with women who experienced mild preeclampsia [4].

According to the World Health Organization, HRQoL is defined as an individual’s perception or concept encompassing mental health status, physical well-being, and psychological well-being [5]. It is also the perception of their position in life in the context of the culture and value systems in which they live and in relation to their goals, expectations, standards, and concerns [5].

Women with preeclampsia suffer from physical discomforts such as headaches, right upper quadrant pain, visual disturbances, and fatigue, with the condition having far-reaching effects on their emotional and psychological well-being [4, 6]. Preeclampsia can negatively affect women’s HRQoL by reducing their mental, physical, social, psychological, and sexual functioning [7]. Thus, improving the HRQoL of postpartum women is the goal of maternal healthcare clinicians, and policy-makers [8].

Common contributing factors impacting maternal HRQoL have been found to include: emotional disturbances, short birth interval, stressful work environment, gender, education, gestational age, advancing age, low income, poor physical health, Khat chewing, lack of social support, personal history of mental illness, unwanted pregnancy, high perceived stress, stillbirth, premature birth, low birth weight, low Apgar scores, personal history of depression, antenatal depression, low self-esteem, and negative cognitive style for maternal HRQoL [4, 5, 7, 9–12].

A study in Ethiopia reported that 62.3% of pregnant women had lower HRQoL, and of these, 46.2% of them had lower physical HRQoL and 79% of them had lower mental HRQoL [13]. This could be related to pregnancy-related complications, sleeping positions, physical pain, and difficulty in falling asleep among postpartum women [7, 14]. Another study identified that the prevalence of perceived stress among pregnant women was 11.6%, which can adversely affect the mother and baby [14]. A study conducted in Ethiopia in 2021 focused on the psychosocial factors of participants and found that 52.6% had poor social support, and 30.4% had moderate social support [15].

The Ethiopian Federal Ministry of Health policy acknowledges the importance of mental health services for the welfare of pregnant women [16]. In 2019, 26% of health facilities had integrated mental health services into their general health services [17]. The shortage of mental health services for a mentally ill postpartum woman contributes to the occurrence of poorer HRQoL [16]. In Ethiopia, a study identified that mental health problems in pregnant and postpartum women ranged from 9.2–33% [16–18], and that 31% of women feared preeclampsia would recur and 11% of them did not want another pregnancy because of this fear, compared to none of the control women reported fear for another pregnancy [16].

Most of the previous research on the impact of preeclampsia on women’s HRQoL in southern Ethiopia has focused on identifying and quantifying problems such as adverse pregnancy outcomes, breastfeeding, family planning issues, and feeding problems [19–21]. However, the findings from these studies do not fully explore the full spectrum of physical, mental, social, and psychological health associated with preeclampsia.

This study aimed to measure the HRQoL and its contributing factors among postpartum women with preeclampsia in the Sidama region, southern Ethiopia. We hypothesised that lower HRQoL is higher among postpartum women with preeclampsia compared to normotensive women. This study will provide epidemiological evidence for the HRQoL among postpartum women with preeclampsia in clinical and public health practices and its contributing factors. This could support efforts to redesign existing postnatal care intervention services.

## Methods

### Study design and setting

A prospective cohort study was conducted from October 2020 to January 2021 in the Sidama region of Ethiopia. In 2020, the population of the region was approximately 4 million. At that time, there were thirteen public hospitals, 138 health centers, and 540 health posts providing maternal, newborn, and child health services. In 2020, approximately 132,031 pregnant women attended ≥4 antenatal care visits (ANC), and 127,585 births were assisted by skilled birth attendants. Out of the 13 hospitals that are found in the region, we enrolled participants from seven of the hospitals, including Adare, Hawassa, Yirgalem, Hula, Bona, Chuko, and Daye hospitals.

### Study population

The participants of this study were women with preeclampsia and normotensive women who were enrolled at ≥20 weeks of gestation until the 37th week. Participants were followed until 12 weeks after delivery. Pregnant women with preeclampsia and normotensive women were selected by health care providers: general medical practitioners, emergency surgical officers, or obstetricians/gynecologists during the follow-up. Pregnant women with hypertension plus proteinuria, mild hypertension and evidence of organ dysfunction, severe hypertensive without proteinuria, and evidence of organ dysfunction were included in the study [1].

### Operational definitions

The diagnosis of preeclampsia was supported by the recent guidelines of the International Society for the Study of Hypertension in Pregnancy [1]. Preeclampsia was defined as the presence of proteinuria (≥1+ or 0.3 g/L) and hypertension (≥140/90 mmHg) on two occasions, at least 4 hours apart, detected after the 20th week of gestation in a previously normotensive woman. Preeclampsia with severe features was defined as one or more of the following conditions: BP ≥ 160/110 mmHg, hepatic dysfunction, pulmonary edema, and/or altered mental status, headache, blurred vision, right upper quadrant pain, blindness, seizures, disseminated intravascular coagulation, and elevated liver enzymes. Gestational age was calculated based on a woman’s recall of her last menstrual period. However, an ultrasound scan was used for those women who could not remember their last menstrual period [1].

### Sample size and sampling

The sample size was calculated using EPI INFO version 7. We could not identify any study conducted in Ethiopia to determine the quality of life of women with preeclampsia in the postpartum period. Therefore, we conducted a tool validation to determine the proportion of women with preeclampsia who had persistence of severity symptoms [22]. We found that the proportion of persistent severity symptoms among postpartum women with preeclampsia was 39.25%, and postpartum women with preeclampsia who did not have persistent severity symptoms were 51.5% [22]. The ratio of the exposed to unexposed group (1 to 1), accounted for a design effect of two and a 10% loss to follow-up. We also assumed a two-sided confidence level of 95% with a power of 80%. Then the sample size was estimated to be 605 women (303 women with preeclampsia and 302 normotensive women).

A two-stage random sampling technique was used to recruit study participants. In the first stage, seven of the thirteen hospitals were selected using a simple random sampling technique. In the second stage, postpartum women with preeclampsia and normotensive women were selected using a simple random sampling technique.

### Data collection

The enrolled women’s HRQoL status was ascertained in the postpartum period at two time points; the 6th and 12th weeks. Before data collection, we validated the data collection tool [22]. Two bilingual translators (speakers of both Sidamic and English languages) were selected to translate the information into the Sidamic language in a way that more accurately reflected the tone of the language. The translations were compared and discrepancies were noted during the translation process. Poorer wording choices were identified and resolved in a discussion between the translators.

The back translations were done by two experts in the source language (English). Face and content validation of the tool was done by a panel of experts (midwife experts, epidemiologists, and gynecologists). The panel of experts independently assessed the tool for readability, intelligibility, clarity, and ease of use. The internal consistency for each dimension was checked using Cronbach’s alpha (Cronbach’s alpha = 0.98) [22].

The tool consisted of two parts. The first part evaluated the individual’s overall perception of HRQoL and the individual’s overall perception of their general health condition. The second part comprised 24 items with four domains, such as physical (7 items), psychological (6 items), social relationships (3 items), and environmental (8 items). In addition, the global scores of general HRQoL and general health condition had one item each [23]. The WHOQOL-BREF tool had five Likert-type response options. The scale ranged from 1 to 5: 1(very poor/very dissatisfied/not at all), 2(poor/dissatisfied/a little), 3 (neither poor nor good/neither satisfied nor dissatisfied/a moderate amount), 4(good/satisfied/very much), and 5(very good/very satisfied/an extreme amount). Each domain was made up of items for which the scores varied between one and five [23]. We computed the scores of four domains by adding the scores noted in each item. A total score was calculated for each domain by adding total item scores divided by the number of items to enable comparison between domains. Assessment of HRQoL of participants was based on total scores on the WHOQoL-BREF. Higher scores on the WHOQoL-BREF reflected a higher HRQoL [23]. Moreover, higher scores on the WHOQoL-BREF reflected better mental health, physical well-being, and psychological well-being of the women.

In the first pilot test, conducted in a non-study area, all participants responded to all items in the data collection tool and marked them correctly. No missing items were found. Data collectors also reported no difficulty in asking the questions, and no participant reported having any problem understanding the items. The tool was tested for the second time 2 weeks after the first measurement. The two-week test-retest reliability result was shown to have a good correlation with reliable strategies to assess these point scores (Intraclass Correlation Coefficients (ICC) for agreement of 0.78; *p <* 0.001) because the ICC value was found to be in the range of 0.75 to 0.9, indicating good reliability [24].

At the two observation points of 6 and 12 weeks, postpartum women with preeclampsia and normotensive women were contacted by midwives from the respective hospitals. The interviews were conducted in pre-arranged quiet rooms in the hospitals where the study participants could complete the interviews undisturbed. The data collection procedures were supervised by three Maternal and Child Health maternity and reproductive health professionals.

The midwives conducted face-to-face interviews at postnatal care clinics using the WHOQOL-BREF tool. Besides, a checklist was used to collect information from the maternal and neonatal records of postpartum women with preeclampsia and normotensive women in each hospital. Similarly, socio-demographic variables such as maternal age, religion, residence, education, and maternal and husband’s occupation were collected.

We collected clinical and laboratory variables linked to postpartum women with preeclampsia and normotensive women through medical records and using a checklist: blood pressure, urine protein, gestational age, number of maternal intensive care unit admissions (ICUs), convulsion, parity, gravidity, and sonographic scan.

### Statistical analysis

The primary outcome variable was the HRQoL. Data were cleaned, coded, and analyzed using Stata 14. We identified outliers and missing values and checked data consistency using the original questionnaire for the responses using participants’ code numbers. Mean and standard deviations were computed for continuous variables. Frequencies and percentages were computed for categorical variables.

Mean and frequency differences were measured through paired-sample t-test, Analysis of Variance (ANOVA), and chi-square statistics. A paired-sample t-test was performed to compare WHOQOL-BREF scores at 6 and 12 weeks in women in the preeclampsia group compared to the normotensive group. An ANOVA was conducted to determine the differences in HRQoL between women in the preeclampsia group and the normotensive group. A chi-squared test was conducted to compare categorical variables between women with preeclampsia and normotensive women in the postpartum period.

Principal component analysis was computed and used for wealth index computation and was ranked into three groups as low, middle, and high. A composite measure of the household’s cumulative living standard was calculated by using data on household ownership of selected assets, like various household assets and means of transportation. Different items for urban and rural areas were computed separately. We included 21 items for rural residents and 16 items for urban residents. The suitability of data was computed by using Bartlett’s test of Sphericity and the Kaiser-Meyer-Olkin (KMO) measure of sample adequacy [25]. The KMO > 0.6 was used to confirm the sample adequacy for factor analysis [25].

A multivariable linear regression analysis was performed to identify contributing factors to HRQoL among postpartum women with preeclampsia and normotensive women. A simple linear regression analysis was performed to identify each contributing factor associated with postpartum HRQoL. According to Hosmer and Lemeshow, a variable with a *p-*value < 0.25 was recommended as a screening criterion for the selection of candidate variables used in a multivariable linear regression model [26]. Contributing factors were reported using the beta-coefficient with a 95% confidence interval. This confirmed that insignificant variables from the first step were reanalyzed in later steps [26]. Moreover, the candidate variables were also considered based on the subject matter expertise of professionals such as gynecologists, obstetricians, epidemiologists, and statisticians who were working as supervisors and who provided more subject matter expertise to improve the modeling process substantially. Indeed, this insight from subject matter experts substantially improved the modeling process [26]. A variable with a *p-*value of < 0.05 was used to identify statistically significant contributing factors to postpartum women’s HRQoL.

We checked for multicollinearity among contributing factors using a variance inflation factor at a cutoff point of ten [27]. We confirmed that there was no collinearity among predictors. The goodness of fit was tested using the Hosmer-Lemeshow test [28]. The predictor that was greater than the significance level (*p-*value > 0.05) was accepted [28]. This indicates that the observed model did not significantly differ from the expected model. A coefficient of determination (R2) was conducted to evaluate how much of the total variation in postpartum HRQoL was explained by all the regression variables.

### Ethical considerations

This study was reviewed and ethically approved by the Institutional Review Board of the University of Gondar R.No: (O/V/P/RCS/044/2019 in March 2019). All participants signed an informed consent document before study participation began. Postpartum women with preeclampsia who have abnormal clinical and laboratory results were referred for treatment. Women with severe hypertension were provided with antihypertensive drugs; those with convulsions were also provided with appropriate treatment.

## Results

### Socio-demographic and economic characteristics of participants

**Fig. 1:**
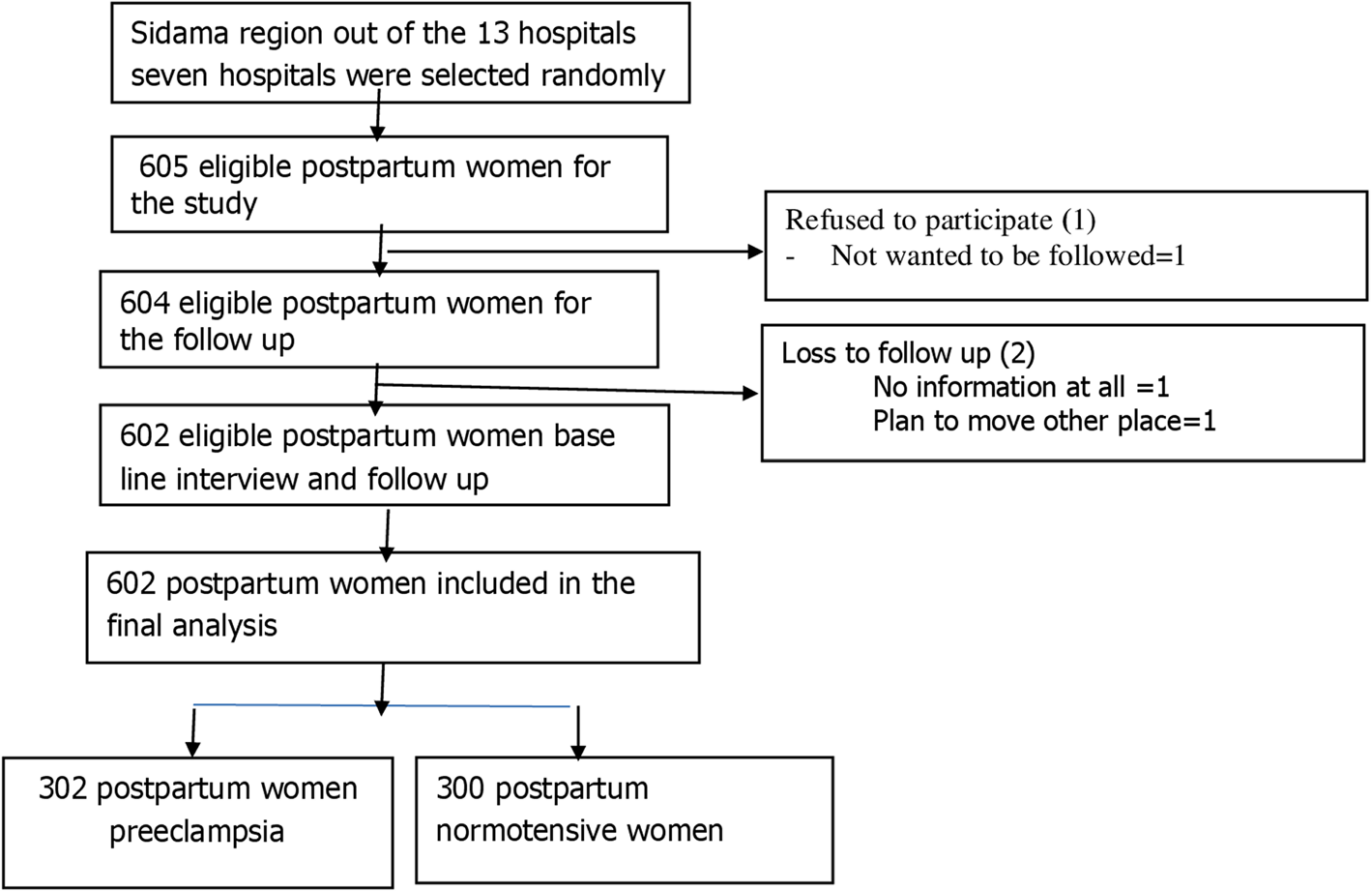
The overall study process in Sidama region southern Ethiopia from October 2020 to January 2021

Of the planned sample size of 605 postpartum women with preeclampsia and normotensive, 602 postpartum women were enrolled, and the analysis was computed. Two (0.33%) of the participants were lost to follow-up. Of these, one was from the preeclampsia group and one from the normotensive group. One participant refused to participate in the study. During the follow-up, eight normotensive women developed preeclampsia. We included these eight women in the preeclampsia group (Fig. 1).

The mean age of the postpartum women with preeclampsia was 24.81 ± 4.47 years, and 24.81 ± 4.46) years for the normotensive group. More than half of the participants were women with preeclampsia (56.6%, 171/302, *p <* 0.05) and were 16–24 years old compared to the normotensive group (42.7%, 128/300). Compared to the normotensive group (37%, 111/300), a higher proportion of postpartum women with preeclampsia was observed in housewife women (46%, 139/302), *p <* 0.05) (Table 1).

**Table 1:** Socio-demographic and economic characteristics of participants

Variables	Preeclampsia (*n =* 302)	Normotensive (*n =* 300)	Total (*n =* 602)	*p*-value[*]
Age of women (year)	Mean ± SD = 24.8 ± 4.47	24.81 ± 4.46		
16–24	171 (56.6)	128 (42.7)	299 (49.7)	*p <* 0.05
25–34	123 (40.7)	156 (52)	279 (46.3)	
> 35	8 (2.6)	16 (5.3)	24 (4)	
Maternal education				
No formal education	17 (5.6)	12 (4)	29 (4.8)	*p* > 0.05
Primary education	132 (43.7)	110 (36.7)	242 (40.2)	
Secondary education	81 (26.8)	97 (32.3)	178 (29.6)	
College/University	72 (23.8)	81 (27)	153 (25.4)	
Husband education				
No formal education	10 (3.3)	4 (1.3)	14 (2.3)	p > 0.05
Primary education	88 (29.1)	81 (27)	169 (28.1)	
Secondary education	88 (29.1)	80 (26.7)	168 (27.9)	
College/University	116 (38.4)	135 (45)	251 (41.7)	
Religion				
Orthodox	47 (15.6)	38 (12.7)	85 (14.1)	*p <* 0.05
Protestant	219 (72.9)	238 (79.3)	457 (75.9)	
Muslim	12 (4)	17 (5.7)	29 (4.8)	
Others (catholic and Jehovah)	24 (7.9)	7 (2.3)	31 (5.1)	
Maternal occupation				
House wife	139 (46)	111 (37)	250 (41.5)	*p <* 0.05
Merchant	63 (20.9)	73 (24.3)	136 (22.6)	
Employed	63 (20.9)	79 (26.3)	142 (23.6)	
Student	21 (7)	23 (7.7)	44 (7.3)	
Farmer	10 (3.3)	7 (2.3)	17 (2.8)	
Daily laborer	6 (2)	7 (2.3)	13 (2.2)	
Husband occupation				
Employed	111 (36.8)	121 (40.3)	232 (38.5)	*p >* 0.05
Merchant	107 (35.4)	104 (34.7)	211 (35)	
Farmer	46 (15.2)	42 (14)	88 (14.6)	
Daily laborer	27 (8.9)	20 (6.7)	47 (7.8)	
Unemployed	11 (3.6)	13 (4.3)	24 (4)	
Wealth index				
Poor	118 (39.1)	101 (33.7)	219 (36.4)	*p >* 0.05
Middle	106 (35.1)	102 (34)	208 (34.6)	
Rich	78 (25.8)	97 (32.3)	175 (29.1)	
Place of residence				
Urban	58 (19.2)	43 (14.3)	101 (16.8)	*p >* 0.05
Rural	244 (80.8)	257 (85.7)	501 (4)	

* = *p-*value of < 0.05 was considered statistically significant

### Obstetric characteristics of participants

Of those women admitted to the hospital at < 34 weeks, a higher proportion had preeclampsia (25.2%, 76/302, *p <* 0.05) compared to the normotensive group (18.3%, 55/300). Compared to the normotensive group (3%, 3/300), a higher proportion of very preterm births (6.6%, 20/302, *p <* 0.05) was observed among women with preeclampsia. A higher proportion of perinatal deaths (14.6%, 44/302, *p <* 0.001) was observed among women with preeclampsia compared to the normotensive group (4%, 12/300) (Table 2).

**Table 2:** Obstetric characteristics of participants

Variables	Preeclampsia (*n =* 302)	Normotensive (*n =* 300)	Total (*n =* 602)	*p*-value[*]
Parity				
Nulliparous	16 (5.3)	9 (3)	25 (4.2)	
1	36 (11.9)	24 (8)	60 (10)	*p >* 0.05
2–3	172 (57)	192 (64)	364 (60.5)	
> 4	78 (25.8)	75 (25)	153 (25.4)	
Gravidity				
1	40 (13.2)	20 (6.7)	60 (10)	
2–3	169 (56)	191 (63.7)	360 (59.8)	*p <* 0.05
> 4	93 (30.8)	89 (29.7)	182 (30.2)	
Mode of delivery				
Spontaneous vaginal delivery	181 (59.9)	162 (54)	343 (57)	*p >* 0.05
Cesarean delivery	106 (35.1)	122 (40.7)	228 (37.9)	
Vacuum assisted delivery	15 (5)	16 (5.3)	31 (5.1)	
Interpregnancy Interval (IPI)				
< 24 months (short IPI)	15 (5)	7 (2.3)	22 (3.7)	
24–59 months (optimal/IPI)	189 (62.6)	192 (64)	381 (63.3)	*p >* 0.05
60+ months (long IPI)	64 (21.2)	68 (22.7)	132 (21.9)	
Not applicable (prim)	34 (11.3)	33 (11)	67 (11.1)	
Gestational age at admission (week)				
< 34	76 (25.2)	55 (18.3)	131 (21.8)	*p <* 0.05
34–37	226 (74.8)	245 (81.7)	471 (78.2)	
Maternal intensive care unit admission				
Yes	7 (2.3)	1 (0.3)	8 (1.3)	*p >* 0.05
No	295 (97.7)	299 (99.7)	594 (98.7)	
Gestational age at delivery (week)				
Extremely preterm (< 28)	6 (2)	7 (2.3)	13 (2.2)	*p <* 0.05
Very preterm (28–32)	20 (6.6)	3 (1)	23 (3.8)	
Moderate to late preterm (32–37)	76 (25.2)	45 (15)	121 (15)	
Term+(> 37)	200 (66.2)	245 (81.7)	445 (73.9)	
Admission to neonatal intensive care unit				
Yes	70 (23.2)	36 (12)	106 (17.6)	*p <* 0.001
No	232 (76.8)	264 (88)	496 (82.4)	
Stillbirth				
Yes	23 (7.6)	5 (1.7)	28 (4.7)	*p <* 0.001
No	279 (92.4)	295 (98.3)	574 (95.3)	
Preterm birth				
Yes	67 (22.2)	25 (8.3)	92 (15.3)	*p <* 0.001
No	235 (77.8)	275 (91.7)	510 (84.7)	
Birth asphyxia				
Yes	30 (9.9)	12 (4)	42 (7)	*p <* 0.05
No	272 (90.1)	288 (96)	560 (93)	
Early neonatal death				
Yes	21 (7)	7 (2.3)	28 (4.7)	*p <* 0.001
No	281 (93)	293 (97.7)	574 (95.3)	
Perinatal death				
Yes	44 (14.6)	12 (4)	56 (9.3)	*p <* 0.001
No	258 (85.4)	288 (96)	546 (90.7)	
Birth weight of newborn (gram)				
< 1500	24 (7.9)	9 (3)	33 (5.5)	
1500–2499	92 (30.5)	21 (7)	113 (18.8)	
2500–3999	176 (58.3)	246 (82)	422 (70.1)	*p <* 0.001
> 4000	10 (3.3)	24 (8)	34 (5.6)	

* = *p-*value of < 0.05 was considered statistically significant

### HRQoL of postpartum women with preeclampsia at 6 and 12 weeks reported by domain

The overall HRQoL improved on WHOQOL-BREF scales from 6 (151 ± 17, *p <* 0.001) to 12 (167 ± 18) weeks in the postpartum period. However, the overall HRQoL scores were significantly lower (*p <* 0.001) among women with preeclampsia compared to normotensive women.

At 6 weeks of the postpartum period, the general HRQoL score was significantly lower among women with preeclampsia (152 ± 41, *p <* 0.001), compared to normotensive women (162 ± 21). Women with preeclampsia had a significantly lower score (146 ± 21, *p <* 0.05) in the physical domain compared to normotensive women (167 ± 14). Women with preeclampsia had a significantly lower score (141 ± 30, *p <* 0.001) on the general health condition compared to normotensive women (171 ± 18). Women with preeclampsia had a significantly lower score (136 ± 22, *p <* 0.001) in the social relationship domain compared to normotensive women (150 ± 21).

At 12 weeks of the postpartum period, the general HRQoL score was significantly lower (163 ± 22, *p <* 0.001) among women with preeclampsia, compared to normotensive women (176 ± 18). Women with preeclampsia had a significantly lower score (166 ± 19, *p <* 0.001) on the general health condition compared to normotensive women (177 ± 16). Women with preeclampsia had a significantly lower score (174 ± 14, *p <* 0.05) in the physical domain compared to normotensive women (191 ± 10). Women with preeclampsia had significantly lower scores (165 ± 26, *p <* 0.001) on psychological well-being compared to normotensive women (173 ± 17). Women with preeclampsia had a significantly lower score (153 ± 23, *p <* 0.001) on social relationships compared to normotensive women (169 ± 12) (Table 3).

**Table 3:** WHOQOL-BREF tool for measuring postpartum Health related Quality of life of women with preeclampsia and normotensive at 6 and 12 weeks reported by domain

Domain/facet	6 weeks postpartum women		12 weeks postpartum women		*p-*value[*]
	Preeclampsia (*n =* 302)	Normotensive (*n =* 300)	Preeclampsia (*n =* 302)	Normotensive (*n =* 300)	
General HRQoL	152 ± 41	162 ± 21	163 ± 22	176 ± 18	*p <* 0.001
General health condition	141 ± 30	171 ± 18	166 ± 19	177 ± 16	*p <* 0.001
Physical domain	146 ± 21	167 ± 14	174 ± 14	191 ± 10	*p <* 0.05
Psychological well-being	150 ± 16	162 ± 12	165 ± 26	173 ± 17	*p <* 0.001
Social relationships	136 ± 22	150 ± 21	153 ± 23	169 ± 12	*p <* 0.001
Environmental domain	169 ± 23	177 ± 19	182 ± 17	195 ± 15	*p >* 0.05
Overall HRQoL	151 ± 17	164 ± 14	167 ± 18	187 ± 14	*p <* 0.001

* **=** *p-*value of < 0.05 was considered statistically significant, *HRQoL* Health related quality of life, *SD* Standard deviation

At 6 weeks of the postpartum period, in the physical domain, women with preeclampsia were more dissatisfied (170 ± 21, *p <* 0.05) with sleep HRQoL compared to normotensive women (147 ± 22). In the social domain, women with preeclampsia who had sexual relationships with partners were more dissatisfied (155 ± 23, *p <* 0.001) compared to normotensive women (143 ± 17). Women with preeclampsia were more dissatisfied (156 ± 19, *p <* 0.001) with social support from friends compared to normotensive women (147 ± 26) [See Table S1 in the supplementary document].

### Contributing factors for postpartum women’s health-related quality of life

In the simple linear regression analysis, the following variables were identified as candidate variables for multivariable linear regression analysis: age of the mother, women with severe features of preeclampsia, preterm birth, birth asphyxia, early neonatal death, birth weight of the newborn, and perinatal death. At 6 weeks of the postpartum period, the physical domain, psychological domain, social domain, and environmental domain, and at 12 weeks of the postpartum period, the physical domain, psychological domain, social domain, and environmental domain were identified as candidate variables for multivariable linear regression analysis.

After controlling for confounders, we identified significant contributing factors for HRQoL, including women with severe features of preeclampsia, preterm birth, and early neonatal death. The physical domain, psychological domain, social domain, and environmental domain were identified as contributing factors for HRQoL at 6 weeks of the postpartum period. At 12 weeks of the postpartum period, the physical domain, psychological domain, and social domain were identified as contributing factors for HRQoL.

Women with severe features of preeclampsia were found to have a significantly higher effect on the lower HRQoL of women with preeclampsia [β = 1.26, 95% CI: 1.11–1.48] compared to women who did not have severe features of preeclampsia. An experience of early neonatal death was found to have a significant negative effect on the HRQoL of women with preeclampsia [β = −2.1, 95% CI: −3.43– −0.85] compared to normotensive women who did not have early neonatal death. An experience of preterm birth was found to have a significant negative effect on HRQoL of women with preeclampsia [β = −1.27, 95% CI: − 2.2– − 0.30] compared to normotensive women who did not experience preterm birth.

At 6 weeks of the postpartum period, the physical domain was found to have a significantly higher effect on the lower HRQoL of women with preeclampsia [β = 1.04, 95% CI: 0.88–1.12] compared to normotensive women, while other factors were constant. While other factors were controlled, social relationships were found to have a significantly higher effect on the lower HRQoL of women with preeclampsia [β = 1.08, 95% CI: 0.90–1.26] compared to normotensive women. When compared to normotensive women, the psychological domain had a significantly higher effect on the lower HRQoL of women with preeclampsia [= 1.93, 95% CI: 0.82–2.03].

At 12 weeks of the postpartum period, the physical domain was found to have a significant contribution to HRQoL of women with preeclampsia [β = 0.99, 95% CI: 0.88–1.10] compared to normotensive women, while other factors were kept constant. When compared to normotensive women, the psychological domain had a significantly higher effect on the lower HRQoL of women with preeclampsia [β = 1.19, 95% CI: 1.09–1.29] compared to normotensive women (Table 4).

**Table 4:** Simple and multivariable linear regression analysis for health-related quality of life of women with preeclampsia and normotensive women

Variables	Unstandardized βeta-coefficient 95% CI	Standardized βeta-coefficient 95% CI
Age of mother (year)		
16–24	−19.58*[− 30– − 8.0]	1.29[− 1.58–2.57]
25–34	− 8.55[− 19.7–2.6]	1.32[− 1.53–2.18]
> 35	1	1
6 weeks postpartum women HRQoL		
Physical domain	4.6***[4.2–4.9]	1.04***[0.88–1.12]
Psychological well-being	4.6***[4.2–4.9]	1.93***[0.82–2.03]
Social relationship	3.9***[3.6–4.2]	1.08***[0.90–1.26]
Environment	6.8***[6.2, 7.4]	1.26***[1.18–3.33]
12 weeks postpartum women HRQoL		
Physical domain	0.87***[3.68–4.2]	0.99***[0.88–1.10]
Psychological well-being	3.6***[3.3–3.9]	1.19***[1.09, 1.29]
Social relationships	6.9***[6.5–7.5]	1.77***[1.57–1.96]
Environment	3.3***[3.1–3.5]	1.42[−2.31–3.40
Women with severe features of preeclampsia		
Yes	1.8[−4.37–8.02]	1.26***[1.11–1.48]
No	1	1
Birth weight of newborn (gram)		
< 1500	−15**[−17– −13]	−1.14[−1.85–1.56]
1500–2499	− 14***[−20– −9]	− 1.16[− 1.68–0.14]
2500–3999	1	1
> 4000	−9.78 [0.69–8.8]	−1.25[− 1.76–1.24]
Preterm birth		
Yes	−13***[− 19– −7]	−1.27**[− 2.2– − 0.30]
No	1	1
Newborn birth asphyxia		
Yes	3.42[−5.17, 12]	−7.48[−15.6–0.65]
No	1	1
Early neonatal death		
Yes	16***[11–18]	−2.1**[−3.43– − 0.85]
No	1	1
Perinatal death		
Yes	2.8**[1.37–5.5]	−4.9[−19–9.17]
No	1	1

*p-value< 0.05, **p-value < 0.001, ***p-value< 0.0001, *CI* Confidence Interval, *β* Beta coefficient, Coefficient of determination (R2) =0.41 = 41%

## Discussion

The HRQoL of women with preeclampsia improved over time from 6 to 12 weeks. However, the overall HRQoL of postpartum women with preeclampsia was significantly lower at 6 and 12 weeks of the postpartum period compared to normotensive women. We identified significant contributing factors for postpartum women’s HRQoL as women with severe features of preeclampsia, preterm birth, and early neonatal death. At 6 weeks of the postpartum period, the physical domain, psychological domain, social domain, and environmental domain were identified as contributing factors to lower HRQoL among women with preeclampsia. At 12 weeks of the postpartum period, the physical domain, psychological domain, and social domain were identified as contributing factors to lower HRQoL among women with preeclampsia.

The HRQoL of women with preeclampsia improved over time from 6 to 12 weeks. This finding agreed with other studies that found HRQoL improved over time from 6 to 12 weeks postpartum [4, 29, 30]. This could be due to a lower physical HRQoL that may continue for up to 6 weeks or more, and a lower mental HRQoL may continue for up to 12 weeks after preeclampsia [30]. Our study results contradict the finding of a study that indicated that, at 12 weeks postpartum, women had recovered mentally, physically, and psychologically, which was reflected in their improving HRQoL [8].

Women with severe features of preeclampsia were found to be significantly associated with lower HRQoL. This finding was similar to other studies that found women with severe features of preeclampsia reported significantly lower HRQoL at 6 weeks of the postpartum period [4, 31]. After severe features of preeclampsia, HRQoL improved for physical/bodily pain, and social functioning. However, some women still reported low mental scores at 12 weeks postpartum, which was associated with the admission of their children to the neonatal intensive care unit or the death of their child [2, 3]. Similarly, women with preeclampsia who reported postpartum depressive symptoms noted they decreased over time from 36% at 6 weeks postpartum to 25% at 12 weeks [4].

Preterm birth showed a negative effect on HRQoL among postpartum women with preeclampsia. This finding was similar to other studies that found preterm birth was found to have a significantly higher effect on a lower HRQoL among postpartum women with preeclampsia compared to women who did not have preterm birth [32, 33]. Furthermore, preterm birth in postpartum women with preeclampsia is associated with feelings of helplessness, fear, and worry about the health of the baby, which may affect postpartum HRQoL [32, 33].

Early neonatal death showed a negative effect on the HRQoL among postpartum women with preeclampsia. This finding was supported by findings of other studies in which early neonatal death was found to be a significant contributing factor to lower mental HRQoL among women with preeclampsia [34, 35]. The same study also showed that early neonatal death was found to have a significant effect on the differences in the prevalence of postpartum depressive symptoms [35].

At 6 weeks of the postpartum period, the general HRQoL was found to be significantly associated with the lower HRQoL of women with preeclampsia compared to normotensive women. This finding was similar to the findings of other studies that women with preeclampsia had significantly lower scores on a general HRQoL, social functioning, emotional role, and mental health [33, 36]. It has been found that women with preeclampsia may experience negative feelings, including hopelessness, guilt, sadness, tearfulness, despair, nervousness, and anxiety [3]. Screening of pregnant women with preeclampsia may lead to a more timely referral and initiation of psychological treatment.

At 6 weeks of the postpartum period, the general health condition was found to be significantly associated with lower HRQoL of women with preeclampsia compared to normotensive women. This was similar to the findings of other studies noting that pregnancy complications are associated with lower physical, mental, and social health [30, 37]. Speed of thinking and clarity of thought, understanding, and concentration might differ among women with preeclampsia and normotensive women in the postpartum period [4]. Furthermore, sleep disturbances seen in pregnancy may be linked to adverse pregnancy outcomes such as cesarean birth and perinatal deaths among postpartum women.

In the social domain, women with preeclampsia were more dissatisfied with personal relationships compared to normotensive women. This was supported by other studies that indicated that women who had a stable relationship with their partners had a higher HRQoL compared with those who did not [13, 37]. This could be because emotions associated with physical challenges during a complicated pregnancy may affect their relationships at home, with friends and partners.

In the social domain, women with preeclampsia were more dissatisfied with social support from friends compared to normotensive women. This finding was similar to findings of other studies that found women with preeclampsia who got support from partners, family, and friends felt supported in their needs, increasing their capacity to address difficult situations favorably [38, 39]. Lower social support negatively affected the physical, social, and environmental well-being of HRQoL. Furthermore, women with severe preeclampsia are dependent on others and seek help in their daily activities. This more likely affects their HRQoL.

In the physical domain, women who had preeclampsia were more dissatisfied with postpartum sleep HRQoL compared to normotensive women. This was consistent with other studies that found deterioration in sleep quality to be a significant issue for postpartum women with preeclampsia because it can impact physiological, cognitive/behavioral, emotional, and social health [38, 39]. This was also supported by other studies that showed that physical and emotional problems can limit postpartum women’s daily activities and affect their HRQoL [40, 41]. This could be further related to preterm delivery-related stress, sleeping position, physical pain, and difficulty in falling asleep.

At 6 weeks of the postpartum period, women with preeclampsia had a lower psychological HRQoL compared to normotensive women. This finding was similar to findings of other studies that reported preeclampsia was considered to have a significant psychological effect on postpartum women, particularly after severe preeclampsia [40, 42]. Postpartum discomfort could be manifested by psychological anxiety, feelings of sadness, problems in the couple’s relationship, and HRQoL [40, 42]. The psychological support provided by healthcare providers should be aware of both childbirth and the prevention of postpartum anxiety and depression.

In the social domain, women with preeclampsia were more dissatisfied with sexual relationships with partners compared to normotensive women. This was similar to findings of other studies that found deterioration of sexual function in women with preeclampsia in the first 6 weeks impairs postpartum HRQoL [42, 43]. This might show women with preeclampsia desired sex and the extent to which the women were able to express and enjoy their sexual desire appropriately. Sexual expression and fulfillment were described without physical intimacy.

In the environmental domain, lower regular leisure time HRQoL scores were observed among women with preeclampsia compared to normotensive women. This finding is similar to findings of other studies that found that regular leisure time for pregnant women was associated with a reduced risk of pregnancy complications such as stress, hypertension, and better mental, and psychological health [41, 44, 45]. Also, it creates a good opportunity for pregnant women to see friends, do sports, or spend time with their families.

In the environmental domain, women who have financial constraints during pregnancy have a lower HRQoL. This was supported by other studies that found women’s financial problems during pregnancy affected their basic needs such as a healthy lifestyle, transportation, and planning for births in health facilities [33, 45]. Furthermore, women with preeclampsia cannot afford to get adequate service, which might affect their HRQoL in the postpartum period.

### Implications for practice

Maternal health care providers should be aware of the potential effect of severe preeclampsia on HRQoL and about the potential need for extended postpartum care of women after severe preeclampsia, especially mental and psychological health care services. Women who have had an early neonatal death or whose children were born prematurely may require extra support. Maternal health care providers should be aware that women with prolonged poor postpartum HRQoL after preeclampsia also experience challenges with work and family responsibilities. Therefore, family members should also be informed about potential mental health issues.

### Limitations

A limitation of our study could be recall bias linked to gestational age, which was calculated based on the women’s recall of her last menstrual period. However, women who could not remember their approximate gestational age were given an ultrasound scan. Social desirability could have been present because data were collected in face-to-face interviews, which could have led to socially acceptable answers. This study is not generalizable as it was limited to one region of the country and those who received hospital care. It also only measured short-term morbidity, so the impact of preeclampsia in extended periods of life, including mental, psychological or other important health outcomes.

## Conclusions

In this study, the health-related quality of life of women with preeclampsia improved over time from 6 to 12 weeks in the postpartum period. However, the overall HRQoL of postpartum women with preeclampsia was significantly lower at both 6 and 12 weeks of the postpartum period compared to normative women. Lower HRQoL was observed among postpartum women with preeclampsia, especially among those who experienced preterm birth, early neonatal death, and women with severe features of preeclampsia. At 6 weeks of the postpartum period, the physical domain, psychological domain, social domain, and environmental domain were identified as contributing factors to lower HRQoL among women with preeclampsia. At 12 weeks of the postpartum period, the physical domain, psychological domain, and social domain were identified as contributing factors to lower HRQoL among women with preeclampsia. The effects of preeclampsia on the HRQoL of postpartum women should be considered in redesigning the existing postnatal care intervention services.

### Implications for future research

This study helps to provide epidemiological evidence for the HRQoL and its contributing factors among postpartum women with preeclampsia in clinical and public health practices. We recommend that early screening of preeclampsia during pregnancy may lead to a more timely referral and initiation of physical, psychological, and social relationship treatment among postpartum women. Furthermore, a large cohort study should be conducted to evaluate other types of hypertensive disorders of pregnancy on the HRQoL of postpartum women.

## Supplementary Information

**Additional file 1: Table S1.** additional results reported by each item in the supplementary document.

## Data Availability

The data that supports the findings of this study is available from the corresponding author upon reasonable request in the form of Stata Version 14.

## Abbreviations

ANC: Antenatal Care Visit
ACOG: American College of Obstetricians and Gynecologists
CI: Confidence Interval
HRQoL: Health Related Quality of Life
HSTP: Health Sector Transformation Plan
VIF: Variation Inflation Factor
WHOQOL-BREF: World Health Organization Quality-of-Life-BREF
