# Supplementary material for: Health-related quality of life among postpartum women with preeclampsia, southern Ethiopia: a prospective cohort study
Source: Health Qual Life Outcomes. 2022 Oct 29;20:147. doi: 10.1186/s12955-022-02061-2 (PMC9618225; doi:10.1186/s12955-022-02061-2)
Supplement: Supplementary file 1 — Additional file 1: Table S1. additional results reported by each item in the supplementary document. [file 12955_2022_2061_MOESM1_ESM.docx]

# Supplementary Material on results reported by each item

# Results

## Table S1: Health related Quality of life of postpartum women with preeclampsia and normotensive at 6 and 12 weeks

| Domains/facet | 6 weeks postpartum | | | 12 weeks of postpartum | | P-value(*) |
| --- | --- | --- | --- | --- | --- | --- |
|  |  | Preeclampsia | Normotensive | preeclampsia | Normotensive |  |
| General QoL | Very poor | 158±19 | 134±20 | 156±17 | 152±41 |  |
|  | Poor | 157±24 | 139±21 | 155±35 | 152±24 |  |
|  | Neither poor nor good | 156±17 | 168±21 | 157±16 | 168±26 | P<0.001 |
|  | Good | 162±19 | 167±22 | 163±16 | 166±19 |  |
|  | Very good | 164±13 | 175±20 | 163±22 | 176±18 |  |
| General health | Very dissatisfied | 155±40 | 154±23 | 152±16 | 153±23 |  |
|  | Dissatisfied | 157±21 | 141±30 | 154±19 | 132±18 |  |
|  | Neither satisfied nor dissatisfied | 156±15 | 171±18 | 138±28 | 171±15 | P<0.001 |
|  | Satisfied | 163±19 | 171±19 | 161±17 | 166±19 |  |
|  | Very satisfied | 170±14 | 175±17 | 161±24 | 177±16 |  |
| Physical domain |  |  |  |  |  |  |
| Items  To what extent do you feel that physical pain prevents you from doing what you need to do? | Not at all | 162±22 | 159±23 | 163±20 | 158±15 |  |
|  | A little | 167±22 | 164±18 | 158±21 | 151±25 |  |
|  | A moderate amount | 161±24 | 159±27 | 162±24 | 163±23 | P<0.05 |
|  | Very much | 157±22 | 163±20 | 162±21 | 167±23 |  |
|  | An extreme amount | 160±21 | 173±21 | 170±22 | 171±14 |  |
| How much do you need any medical treatment to function in your daily life? | Not at all | 154±25 | 174±14 | 165±18 | 167±21 |  |
|  | A little | 165±18 | 166±20 | 153±24 | 156±21 |  |
|  | A moderate amount | 161±20 | 163±15 | 162±22 | 163±20 | P<0.05 |
|  | Very much | 167±22 | 160±25 | 160±22 | 156±25 |  |
|  | An extreme amount | 169±26 | 160±11 | 161±22 | 168±21 |  |
| How well are you able to get around? | Not at all | 158±21 | 154±18 | 159±23 | 148±19 |  |
|  | A little | 160±20 | 155±21 | 163±20 | 160±18 |  |
|  | A moderate amount | 164±21 | 151±19 | 164±23 | 178±18 | P<0.05 |
|  | Very much | 165±19 | 171±19 | 160±19 | 163±13 |  |
|  | An extreme amount | 159±31 | 172±23 | 167±14 | 174±20 |  |
| Do you have enough energy for everyday life | Not at all | 155±23 | 143±31 | 151±21 | 152±29 |  |
|  | A little | 161±17 | 147±27 | 163±18 | 142±20 |  |
|  | Moderately | 167±17 | 156±18 | 154±27 | 156±19 | P<0.05 |
|  | Mostly | 163±20 | 170±25 | 169±19 | 163±12 |  |
|  | Completely | 156±31 | 178±17 | 170±21 | 178±17 | P<0.05 |
| How satisfied are you with your sleep | Very dissatisfied | 159±19 | 145±31 | 156±20 | 142±24 |  |
|  | Dissatisfied | 170±21 | 147±22 | 154±22 | 151±19 |  |
|  | Neither satisfied nor dissatisfied | 163±21 | 155±27 | 142±20 | 171±23 | P<0.05 |
|  | Satisfied | 163±20 | 166±24 | 163±15 | 164±19 |  |
|  | Very satisfied | 155±21 | 173±17 | 166±22 | 176±16 |  |
| How satisfied are you with your ability to perform your daily living activities | Very dissatisfied | 156±22 | 141±22 | 145±16 | 157±20 |  |
|  | Dissatisfied | 159±21 | 148±20 | 151±20 | 157±20 |  |
|  | Neither satisfied nor dissatisfied | 168±19 | 159±17 | 146±21 | 163±21 | P<0.001 |
|  | Satisfied | 165±19 | 173±23 | 162±22 | 165±13 |  |
|  | Very satisfied | 177±11 | 180±17 | 166±22 | 180±14 |  |
| How satisfied are you with your capacity for work? | Very dissatisfied | 163±21 | 141±19 | 156±24 | 155±23 |  |
|  | Dissatisfied | 156±22 | 147±20 | 158±20 | 152±25 |  |
|  | Neither satisfied nor dissatisfied | 155±18 | 167±15 | 150±21 | 161±22 | P<0.001 |
|  | Satisfied | 169±25 | 162±19 | 164±14 | 167±21 |  |
|  | Very satisfied | 170±16 | 180±18 | 163±22 | 177±16 |  |
| Psychological Domain |  |  |  |  |  |  |
| Items | Not at all | 153±21 | 136±21 | 155±22 | 157±23 |  |
| How much do you enjoy life | A little | 159±20 | 128±21 | 156±21 | 136±18 |  |
|  | A moderate amount | 151±17 | 167±17 | 155±20 | 161±18 | P<0.001 |
|  | Very much | 162±17 | 172±20 | 163±13 | 166±21 |  |
|  | An extreme amount | 164±28 | 179±21 | 163±24 | 178±15 |  |
| To what extent do you feel your life to be meaningful | Not at all | 156±20 | 146±33 | 160±21 | 158±21 |  |
|  | A little | 146±24 | 160±21 | 161±18 | 148±25 |  |
|  | A moderate amount | 164±24 | 161±16 | 154±21 | 155±15 | P<0.001 |
|  | Very much | 167±19 | 165±18 | 163±21 | 161±14 |  |
|  | An extreme amount | 170±21 | 177±17 | 166±24 | 175±16 |  |
| How well are you able to concentrate | Not at all | 151±29 | 160±20 | 156±24 | 156±30 |  |
|  | Slightly | 159±20 | 149±24 | 158±20 | 157±26 |  |
|  | A moderate amount | 163±21 | 157±22 | 163±20 | 156±21 | P<0.05 |
|  | Very much | 162±20 | 175±17 | 162±18 | 163±17 |  |
|  | Extremely | 162±20 | 163±29 | 166±23 | 168±17 |  |
| Are you able to accept your bodily appearance | Not at all | 160±20 | 151±29 | 160±19 | 154±23 |  |
|  | Slightly | 159±20 | 149±24 | 158±20 | 146±24 |  |
|  | A moderate amount | 163±21 | 157±22 | 154±15 | 167±20 | P<0.05 |
|  | Very much | 175±17 | 162±20 | 174±18 | 161±17 |  |
|  | Extremely | 163±29 | 162±20 | 157±37 | 176±22 |  |
| How satisfied are you with yourself | Very dissatisfied | 152±22 | 142±23 | 156±20 | 142±23 |  |
|  | Dissatisfied | 159±20 | 136±27 | 156±23 | 147±21 |  |
|  | Neither satisfied nor dissatisfied | 169±16 | 151±14 | 162±22 | 160±29 | P<0.05 |
|  | Satisfied | 178±17 | 163±17 | 164±21 | 163±13 |  |
|  | Very satisfied | 170±24 | 179±17 | 168±21 | 176±17 |  |
| How often do you have negative feelings such as blue mood, despair, anxiety, depression | Never | 165±17 | 168±21 | 170±19 | 167±16 |  |
|  | Seldom | 162±21 | 166±22 | 154±25 | 147±28 |  |
|  | Quite often | 162±20 | 167±24 | 158±18 | 157±20 | P<0.05 |
|  | Very often | 161±21 | 140±25 | 160±23 | 152±23 |  |
|  | Always | 161±21 | 150±24 | 163±19 | 179±20 |  |

HRQoL-Health related Quality of Life, SD-Standard deviation,*= p value<0.05 was considered statistically significant

## Table S1: Health related Quality of life of postpartum women with preeclampsia and normotensive women at 6 and 12 weeks......Continued

| Domains/facet | 6 weeks postpartum | | | 12 weeks postpartum | | P-value (*) |
| --- | --- | --- | --- | --- | --- | --- |
|  |  | PE | Norm | PE | Norm |  |
| Social relation ships |  |  |  |  |  |  |
| Items | Very dissatisfied | 152±22 | 148±21 | 154±25 | 147±24 |  |
| How satisfied are you with your personal relationships | Dissatisfied | 159±19 | 136±25 | 161±20 | 143±14 | P<0.001 |
|  | Neither satisfied nor dissatisfied | 176±15 | 149±19 | 162±16 | 150±22 |  |
|  | Satisfied | 174±22 | 165±15 | 164±23 | 166±14 |  |
|  | Very satisfied | 179±16 | 181±16 | 165±21 | 177±19 |  |
| How satisfied are you with your sex life | Very dissatisfied | 153±28 | 141±25 | 157±24 | 143±26 | P<0.001 |
|  | Dissatisfied | 155±23 | 143±17 | 155±21 | 140±17 |  |
|  | Neither satisfied nor dissatisfied | 156±18 | 168±16 | 164±20 | 161±17 |  |
|  | Satisfied | 168±18 | 176±15 | 167±20 | 169±14 |  |
|  | Very satisfied | 168±32 | 181±15 | 160±26 | 176±20 |  |
| How satisfied are with the support you get from your friends | Very dissatisfied | 158±22 | 155±22 | 159±22 | 146±27 |  |
|  | Dissatisfied | 156±19 | 147±26 | 153±18 | 137±16 | P<0.001 |
|  | Neither satisfied nor dissatisfied | 169±17 | 152±14 | 157±14 | 162±20 |  |
|  | Satisfied | 167±15 | 177±22 | 164±22 | 166±16 |  |
|  | Very satisfied | 176±16 | 186±14 | 167±23 | 177±18 |  |
| Environment |  |  |  |  |  |  |
| Items  How safe do you feel in your daily life | Not at all | 159±22 | 146±27 | 142±25 | 158±22 |  |
|  | Slightly | 153±18 | 137±16 | 148±20 | 152±22 |  |
|  | A moderate amount | 162±20 | 157±14 | 155±19 | 165±23 | P>0.05 |
|  | very much | 164±22 | 166±16 | 165±20 | 163±17 |  |
|  | Extremely | 167±23 | 177±18 | 164±22 | 177±18 |  |
| How healthy is your physical environment | Not at all | 156±21 | 141±30 | 148±22 | 157±19 |  |
|  | Slightly | 150±27 | 158±18 | 160±20 | 145±21 |  |
|  | A moderate amount | 159±16 | 165±23 | 162±24 | 161±17 | P>0.05 |
|  | very much | 163±17 | 171±22 | 164±21 | 163±17 |  |
|  | Extremely | 162±23 | 180±19 | 166±21 | 180±18 |  |
| Have you enough money to meet your needs | Not at all | 153±22 | 121±23 | 158±21 | 135±23 |  |
|  | A little | 161±20 | 156±22 | 159±24 | 158±19 |  |
|  | Moderately | 165±23 | 163±18 | 163±21 | 162±17 | P<0.05 |
|  | Mostly | 166±17 | 167±19 | 171±18 | 168±20 |  |
|  | completely | 170±17 | 165±20 | 162±19 | 182±19 |  |
| How available to you is the information that you need in your daily-to-day life | Not at all | 144±24 | 122±29 | 150±21 | 134±24 |  |
|  | A little | 163±18 | 148±17 | 163±21 | 148±20 | P>0.05 |
|  | Moderately | 167±19 | 164±17 | 164±23 | 166±16 |  |
|  | Mostly | 174±18 | 165±20 | 165±19 | 167±14 |  |
|  | Completely | 160±21 | 177±20 | 167±20 | 181±17 |  |
| To what extent do you have the opportunity for leisure activities | Not at all | 156±20 | 146±33 | 159±20 | 146±30 |  |
|  | A little | 160±21 | 146±24 | 159±21 | 141±22 | P<0.05 |
|  | Moderately | 164±24 | 161±16 | 161±14 | 163±22 |  |
|  | Mostly | 165±18 | 167±19 | 164±22 | 168±18 |  |
|  | Completely | 170±21 | 177±17 | 167±21 | 177±23 |  |
| How satisfied are you with the condition of your living place | Very dissatisfied | 158±19 | 142±19 | 157±19 | 130±17 |  |
|  | Dissatisfied | 158±22 | 147±28 | 153±23 | 141±21 | P>0.05 |
|  | Neither satisfied nor dissatisfied | 157±18 | 169±14 | 155±22 | 164±18 |  |
|  | Satisfied | 170±22 | 165±17 | 165±21 | 166±13 |  |
|  | Very satisfied | 179±15 | 177±19 | 164±28 | 181±18 |  |
| How satisfied are you with your access to health service | Very dissatisfied | 162±21 | 145±28 | 156±23 | 153±36 |  |
|  | Dissatisfied | 159±19 | 136±21 | 153±22 | 139±18 | P<0.05 |
|  | Neither satisfied nor dissatisfied | 160±20 | 158±17 | 151±19 | 162±21 |  |
|  | Satisfied | 165±15 | 173±21 | 163±14 | 166±18 |  |
|  | Very satisfied | 158±30 | 175±18 | 159±22 | 166±22 |  |
| How satisfied are you with your transport | Very dissatisfied | 148±21 | 135±25 | 158±20 | 132±18 |  |
|  | Dissatisfied | 144±20 | 145±25 | 158±21 | 160±21 |  |
|  | Neither satisfied nor dissatisfied | 160±16 | 173±19 | 160±17 | 169±20 | P<0.05 |
|  | Satisfied | 167±17 | 175±18 | 163±19 | 165±15 |  |
|  | Very satisfied | 156±27 | 175±18 | 160±23 | 163±25 |  |

PE=Preeclampsia. Norm=Normotensive, *= p value<0.05 was considered statistically significant
